# Madden–Julian Oscillation Enhances Phytoplankton Biomass in the Maritime Continent

**DOI:** 10.1038/s41598-019-41889-5

**Published:** 2019-04-01

**Authors:** Chiung-Wen June Chang, Huang-Hsiung Hsu, Wee Cheah, Wan-Ling Tseng, Li-Chiang Jiang

**Affiliations:** 1 Department of Atmospheric Sciences, Chinese Cultural University, 114 Taipei, Taiwan; 2 Research Center for Environmental Changes, Academia Sinica, 115 Taipei, Taiwan; 3 Institute of Ocean and Earth Sciences, University of Malaya, 50603 Kuala Lumpur, Malaysia

## Abstract

In addition to monsoon-driven rainfall, the Maritime Continent (MC) is subject to heavy precipitation caused by the Madden–Julian Oscillation (MJO), a tropical convection-coupled circulation that propagates eastward from the Indian to the Pacific Ocean. This study shows that riverine runoff from MJO-driven rainfall in the western MC significantly enhances phytoplankton biomass not only in the coastal regions but as far as the nutrient-poor Banda Sea, located 1,000 km downstream of the riverine source. We present observational estimates of the chlorophyll-a concentration in the Banda Sea increasing by 20% over the winter average within an MJO life cycle. The enhancement of phytoplankton in the central Banda Sea is attributed to two coinciding MJO-triggered mechanisms: enhanced sediment loading and eastward advection of waters with high sediment and chlorophyll concentrations. Our results highlight an unexpected effect of MJO-driven rainfall on the downstream oceanic region. This finding has significant implications for the marine food chain and biogeochemical processes in the MC, given the increasing deforestation rate and projections that global warming will intensify both the frequency and strength of MJO-driven rainfall in the MC.

## Introduction

**Figure 1:**
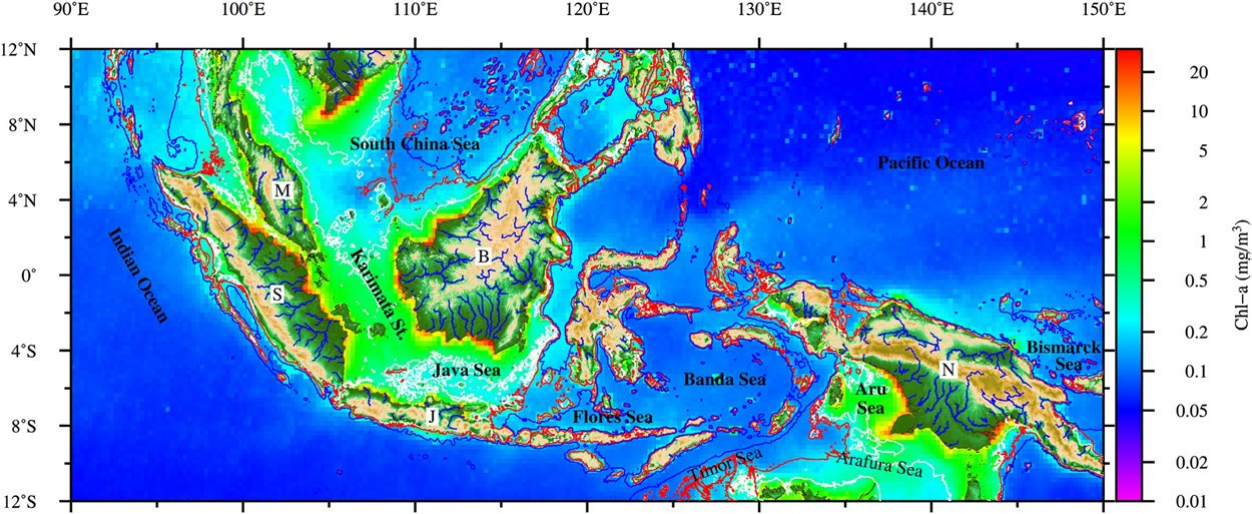
Topography and winter surface chlorophyll-a concentration (December–February average) in the Maritime Continent. Major rivers are indicated by blue lines. Alphabetic labels on major islands: S = Sumatra; M = Malay Peninsula; B = Borneo; N = New Guinea. The −50 m bathymetry layer is marked by a red contour line, and the −100 m layer by a blue line. Chl concentration is high in coastal and shallow areas (<50 m) but much lower in the deep ocean, with a difference of up to two orders of magnitude. This figure was created using Generic Mapping Tools (Ver. 5.4.2). http://gmt.soest.hawaii.edu/projects/gmt.

Surrounded by a warm ocean, the Maritime Continent (MC) is a “wet tropical” region characterized by high precipitation and runoff. A quarter of the total annual sediment exported from land to the global ocean comes from the MC^1,2^; this discharge leads to large nutrient flux and enables high productivity in MC coastal and shelf regions (Fig. 1). Terrestrial nutrients, however, are mostly consumed in the shelf waters before reaching the larger area of the open ocean^3^. In deep MC waters such as the Banda Sea, biological processes are linked to changes in the physical environment associated with climate factors. The surface chlorophyll-a (Chl) concentration in the Banda Sea exhibits strong seasonality in response to monsoon climate forcing^4,5^. Wind-induced vertical mixing and upwelling raise the productivity of this region during the southeast monsoon in the boreal summer. During the northwest monsoon in the boreal winter, however, the Banda Sea becomes oligotrophic because the wind induces downwelling and suppresses the entrainment of nutrients from deep water. By contrast, shallow seas such as the Karimata Strait, Java Sea, and Aru Sea are constantly enriched with nutrients.

**Figure 2:**
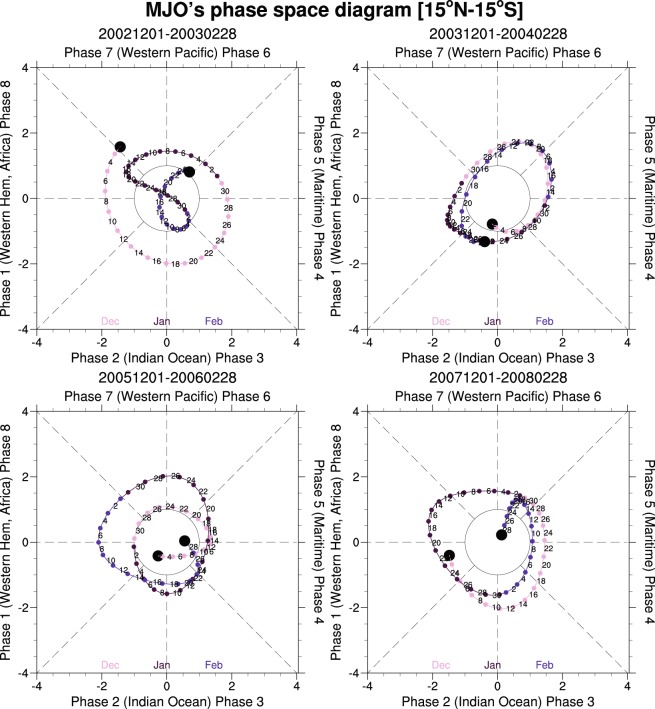
Phase diagrams of strong MJOs occurring between December 2002 and February 2003, December 2003 and February 2004, December 2005 and February 2006, and December 2007 and February 2008. Colored points in the diagrams (representing sequential days) distinguish the different months. The sequential days trace anticlockwise circles around the origin, signifying eastward propagation of the MJO. Distance from the origin is proportional to MJO strength. The amplitude circle in the center of the diagram signifies one-standard-deviation MJO strength.

One of the most prominent meteorological phenomena in the tropics is the eastward-propagating Madden–Julian Oscillation **(**MJO)^6,7^, a subseasonal coupled convection–circulation system with a 30–60-day cycle. Similar to monsoonal forcing, the MJO is characterized by reversals in surface wind direction and distinct wet/dry phases and causes significant fluctuations in surface heat flux, precipitation, and momentum. The precipitation and momentum fluctuations lead to an increase in rainfall, wind-induced surface currents, local entrainment, and ocean surface dynamics during the convection-active phase^8–15^. To date, only a few studies have investigated the effect of the MJO on ocean biology; those few have found that local wind-mixing nutrient entrainment is enhanced in the oceans surrounding the MC^16–20^. However, the biological response to the MJO in island-enclosed waters in the MC has not been investigated. The MC has a relatively complex topography and orography, which complicates interaction of the MJO with the MC^21^. Herein, we report how a distinct process—namely, MJO-driven precipitation and oceanic advection—can greatly affect the Chl concentration in the Banda Sea. This analysis focuses on four extremely strong and long-lasting MJO events during northwest monsoons (December–February) from 2002 to 2010. The MJO phase diagram in Fig. 2 shows that in each of these four events, the oscillation had large amplitude throughout the entire winter season. The characteristics of the four events are also identified in the composites of all MJO events in 2002–2010 provided in the supplementary information online (Supplementary Figs S3–S9).

## Results

### Propagation and footprint of the MJO in the MC

**Figure 3:**
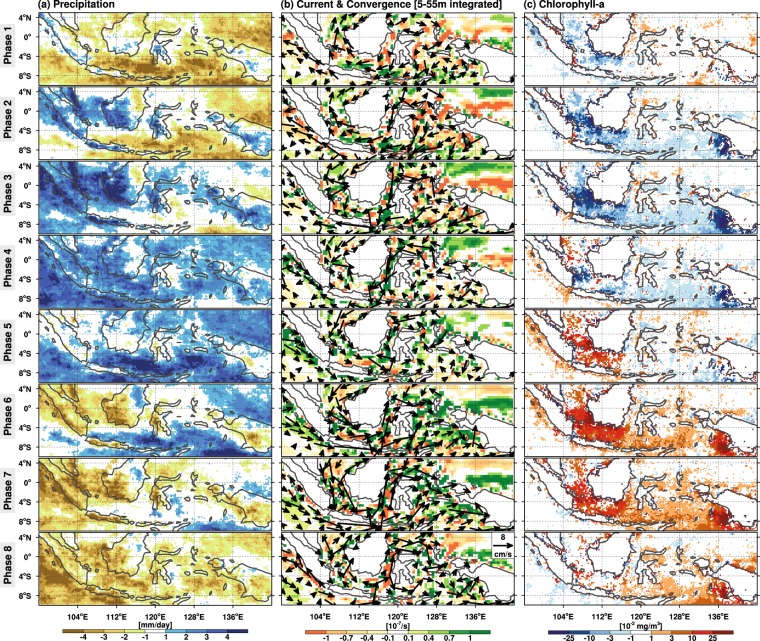
Variable anomalies associated with the eight MJO phases: (**a**) surface precipitation; (**b**) surface-water convergence (shading) overlaid with ocean surface currents (vectors); and (**c**) Chl concentration. Positive (negative) values indicate evidence of enhanced (suppressed) variables during the given phase. Only patterns significant at the 0.05 level are plotted. Surface-water convergence and surface currents in (**b**) are an integrated value over 5–55 meters. This figure was created using the NCAR Command Language (Version 6.3.0) [Software] (2018). Boulder, Colorado: UCAR/NCAR/CISL/TDD. 10.5065/D6WD3XH5.

Analysis of the MJO reveals a large-scale envelope with leading low-level moistening in the initial phase, followed by maximum precipitation in the deep convection region and a trailing wake of upper troposphere stratiform precipitation^22–25^. The envelope also contains surface westerly wind anomalies to the west and easterly wind anomalies to the east of the deep convection core. The eight MJO phase maps in Fig. 3a and Supplementary Fig. S1a illustrate the propagating structures of MJO precipitation and surface wind anomalies, respectively. Anomalous precipitation and westerly winds on the west coast of Sumatra in phases III and IV signal the MJO’s arrival in the MC. From phases V to VIII, the MJO system propagates though the MC, indicated by enhanced westerlies following the maximum rainfall in the narrow oceanic channel, moving from the Karimata Strait through the Java, Flores, Banda, and Arafura Seas to the western Pacific. Before the MJO convection center propagates into the MC, rain is more over land in the MC (the so-called “vanguard precipitation” as mentioned in Peatman *et al*.^26^). Therefore, the western MC enters wet MJO periods in phases II, starting with significant rainfall over the mountainous Malay Peninsula, Sumatra, Java, and Borneo.

The MJO generates significant fluctuations in surface ocean dynamics (Fig. 3b). During phases V–VIII, the onset of southeastward/eastward flow over the oceans follows the eastward spread of westerly winds from the Karimata Straits through the Java Sea to the Banda Sea. Meanwhile, the westerly winds force an Ekman downwelling (surface-water convergence) response within the oceanic channels. Reversed wind and surface ocean flow, and divergence occur during phases I–IV. A consistent phase-dependent modulation of Chl concentration by the MJO is shown in Fig. 3c. Small patches of high-Chl-concentration waters start appearing north of Sumatra in phase IV, then expand over the entire western South China Sea off the Malay Peninsula. These high-Chl-concentration waters reach the Sunda Shelf (an area between the Malay Peninsula, Sumatra, Borneo, and Java) in phase V and then extend to the Banda and Arafura Seas in the following phases (VI–VIII). In phase VIII, high-Chl-concentration anomalies shift further east to the Bismarck Sea east of New Guinea, while Chl concentrations at the Sunda Shelf are considerably reduced. A coherent variation pattern of total suspended matter (TSM), a measure of the concentration of fine-scale debris in surface water, is shown in Supplementary Fig. S1b. Synchronized propagation of Chl and TSM follows the convective phases of the MJO from the southern South China Sea to the Bismarck Sea.

During phases V–VIII, anomalously high Chl and TSM concentrations are observed at the Sunda Shelf and in the Timor, Aru, and Arafura Seas, where the water depth is less than 50 meters (Fig. 1). Although in shallow waters the amount of TSM might be increased through resuspension or mixing by high winds, the anomalously high concentrations of TSM in coastal waters can be attributed to increased terrestrial or river runoff. The release of nutrients from fluvial particles through desorption or remineralization contributes to the available nutrients. As a result, highly nutrient-laden river runoff waters can enhance biological production or, in the worst-case scenario, cause eutrophication in the estuary and coastal areas^27–30^. This observation is consistent with previous findings that large annual sediment discharges from Borneo, the Indonesian island chains, and New Guinea occur surrounding the MC’s shallow waters^1,2^ and cause the fluctuation of TSM in the waters. The MJO-induced land precipitation perturbation around the Sunda Shelf (4°N–3°S, 102°E–115°E) can increase by 30% during the wet phases of the MJO cycle (Table 1) and also considerably elevate the volume of river runoff with a lag time of several days. In general, the peak MJO-related land precipitation in the western MC (as in phases II–IV, Fig. 3a) precedes the TSM maximum at the Sunda Shelf (phases V –VII) by one or two phases. The shelf areas are highly dynamic regions affected by the interaction of winds, waves, and the currents^31,32^. High TSM waters spread further offshore following the MJO convection can be explained in terms of oceanic dynamics as follows. When the MJO westerlies arrive in phase V after the passage of deep convection and precipitation, wind-driven eastward flow leads to further eastward transport of high TSM at the Sunda Shelf. This interpretation is consistent with the simulation results of Ningsih *et al*.^30^, in which they found eastward flow in the Java Sea in the westerly monsoon period whereas westward flow in the easterly monsoon. Other physical processes, such as mixing and resuspension on the shelf, may also be strengthened by the enhanced surface westerly winds and currents although their contribution cannot be evaluated in this study. These processes associated with the westerly wind anomalies, which trail behind the deep convection in an MJO event, can explain the delayed response of TSM maximum at the Sunda Shelf (Supplementary Fig. S1).

**Table 1:** Increment and the increase rate for the anomalous precipitation over land (4°N–3°S,102°E–115°E), the zonal current in the Karimanta Strait (4°S–6°S,108°E–110°E), and the Chl in the Banda Sea (4°S–7°S,125°E–132°E).

Increments [each phase] & Increase rate [each phase/DJF mean]									
Phase	1	2	3	4	5	6	7	8	mean
**Variable**									
Precipitation over land (mm/day) (4°N–3°S,102°E–115°E)	−0.81	2.82	3.5	2.5	0.05	−2.7	−3.5	−2.4	10.79
	−7.5%	26.1%	32.4%	22.7%	0.4%	−25.2%	−32%	−22.4%	
CFSR U current 5–55 m integrated (m/s) (4°S–6°S,108°E–110°E)	−0.025	−0.022	−0.016	0.005	0.029	0.026	0.032	−0.011	0.21
	−11.7%	−10.6%	−7.5%	2.2%	13.6%	12.4%	15.2%	−5.4%	
CFSR U current at 15 m (m/s) (same as above)	−0.018	−0.029	−0.021	−0.006	0.019	0.035	0.029	0.002	0.264
	−7%	−10.8%	−8%	−2.2%	7.3%	13.2%	10.9%	0.6%	
OSCAR U current at 15 m (m/s) (same as above)	−0.0338	−0.0335	−0.036	−0.014	0.028	0.051	0.036	0.023	0.307
	−11%	−10.9%	−11.8%	−4.6%	9.1%	16.6%	11.8%	7.5%	
Chlorophyll-a (10^−2^ mg/m^3^) (4°S–7°S,125°E–132°E)	−0.04	−1.82	−2.4	−1.47	−0.02	2.53	2.07	1.62	12.41
	−0.3%	−14.7%	−19.4%	−11.8%	−0.2%	20.4%	16.7%	13%	

### TSM and Chl in offshore regions

**Figure 4:**
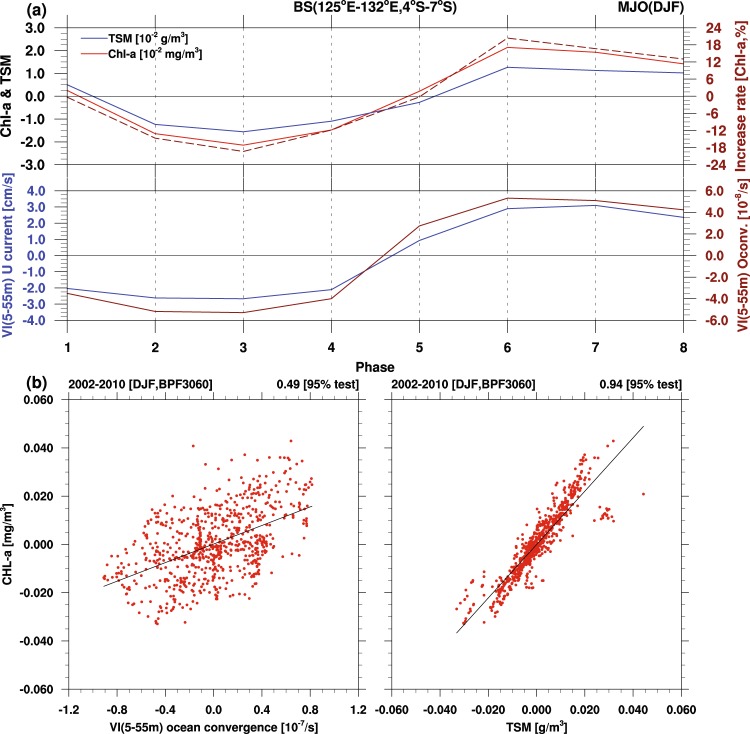
(**a**) Domain-averaged anomalous value of Chl (blue line), TSM(red line), and Chl increase rate (dash line) over the Banda Sea (between 126°–136°E) in the eight MJO phases; (**b**) scatter plots of Chl versus integrated surface-water convergence (left) and TSM (right), based on the daily anomalous value during winter in 2002–2010 (total of 720 days). All correlation coefficients are significant at the 0.05 level.

**Figure 5:**
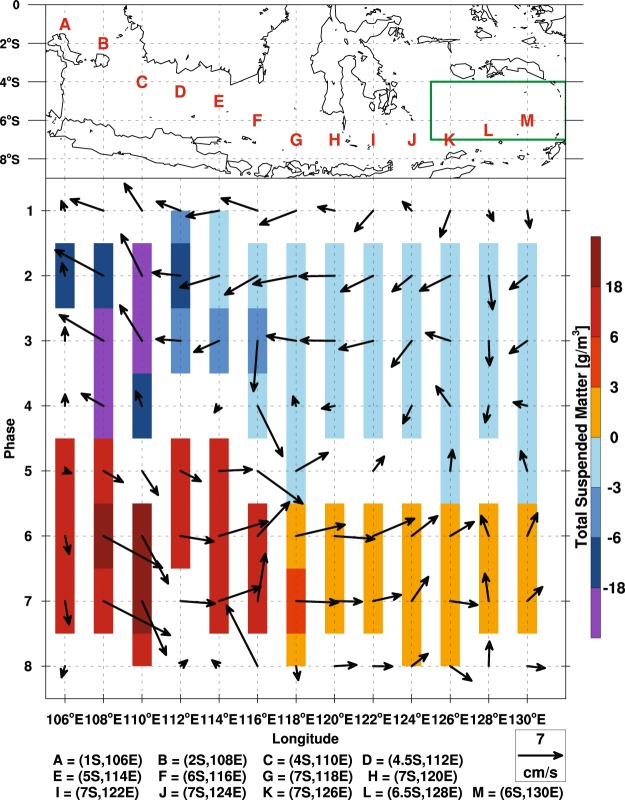
Time series for magnitude and direction of anomalous ocean surface flow (vectors) and TSM (shading) at the upper 5–55 meter level. Marked grid points are spaced at two longitudes, starting from 110°E and moving along the island-surrounded sea channels such as the Karimata Strait, Java Sea, and Flores Sea and down to the Banda Sea. The Banda Sea region (126°–136°E) is marked by the green rectangle.

High concentrations of TSM and Chl appear further offshore in deeper waters such as the Flores Sea and open Banda Sea during phases VI and VII, although the anomalies are relatively less pronounced in comparison to those in the shelf regions. The relationships between Chl/TSM and various atmospheric and oceanic variables in the eight MJO phases are illustrated in Fig. 4a, which shows the domain-averaged anomalous value over the Banda Sea (126°–136°E, 4°–7°S; marked by the rectangle in Fig. 5). Chl fluctuates within an MJO life cycle, with a positive anomaly value that starts rising in phase V, peaks during phases VI and VII, and declines into negative values during phases II–IV. Chl reaches its maximum concentration in phase VI, with a 20% increase relative to the boreal winter mean (dashed line in Fig. 4a, upper panel). Chl is again found to fluctuate simultaneously with TSM, indicating a close relationship between the two variables. Notably, TSM concentrations are three orders of magnitude higher than those of Chl. Because light is the main driver in photosynthesis, we also examined the relationship between irradiance and Chl concentration. In the MJO’s convection-active phases when Chl blooms, photosynthetically available radiation levels are lower because of cloudiness (results not shown). It follows that light availability is not the key controlling factor for Chl bloom in the Banda Sea.

Figure 4a also reveals that positive Chl and TSM anomalies are concurrent with anomalous eastward ocean flow as well as surface-water convergence/downwelling and, conversely, negative Chl and TSM anomalies are concurrent with anomalous westward ocean flow as well as divergence/upwelling. In general, downwelling is associated with a depressed thermocline^33,34^, inhibiting the entrainment of nutrient-rich waters from below. Focusing on the open waters of the Indian Ocean, Jin *et al*.^18^ applied MJO-like forcing to a biological model and suggested that entrainment driven by increased surface winds would cause nutrients to raise up from their normal depth. However, the observed MJO-induced surface downwelling should aggravate the already oligotrophic condition of the winter Banda Sea. The scatter diagrams in Fig. 4b are plotted for surface-water convergence over the ocean and for TSM versus Chl over the Banda Sea, based on subseasonal daily anomalies during 2002–2010. Chl variation is strongly correlated with TSM variation (coefficient = 0.94) and moderately correlated with surface-water convergence (coefficient = 0.46). The strong correlation between Chl and TSM in the Banda Sea indicates that Chl enhancement is mainly a result of high TSM concentration. We argue that enhanced flow convergence would counteract the effect of mixing that is associated with the westerly anomaly and that the Banda Sea’s nutrient-poor deep waters, with a depth exceeding 5,000 meters, are not a favorable environment for Chl enhancement through vertical mixing.

Because it is restricted to coastal and shelf areas, the high TSM concentration occurring in the offshore Banda Sea originated elsewhere, given that the deep central Banda Sea has no proximity to land. The wind-driven flow over the oceans trailing the deep convection eastward of the MJO and the notably higher TSM concentration at the Sunda Shelf indicate that the nutrient- and chlorophyll-enriched waters at the Sunda Shelf are probably transported eastward by wind-driven surface flow. To explore upstream TSM as a contributing factor, a time series of surface flow and TSM concentration along island-surrounded sea channels such as the Karimata Strait, Java Sea, and Flores Sea and down to the Banda Sea is shown in Fig. 5, with eight grid points for every two longitudes starting from 110°E. A dominant eastward component of the surface flow progressively expands from the Karimata Strait to the Banda Sea in phases V–VIII and is followed by the occurrence of high TSM concentrations (indicated by shading). During this period, the zonal component of the wind-driven surface flow speed increases by 15% relative to the mean current (Table 1). This flow tendency changes to westward in phases I–IV, while the direction of the MJO wind becomes easterly. After the eastward MJO wind–driven flow attains its maximum value, the eastward shift of nutrient-loaded waters at the Sunda Shelf boosts phytoplankton growth in the Banda Sea in phases VI–VII (Fig. 4a, Table 1). Because the TSM maximum concentration at the Sunda Shelf is associated with terrestrial nutrient runoffs from MJO precipitation over the Malaysian and Indonesian islands (during phases I–IV), our results establish a direct link between upstream land rainfall and downstream ocean biological response on an MJO timescale. No significant vertical nutrient input from deeper waters is expected when the water mass travels eastward because MJO wind–driven downwelling is observed.

## Summary and Discussion

The availability of nutrients in the tropical ocean is a key factor affecting the growth of phytoplankton. Herein, we used satellite observations and reanalysis data to examine the biological footprint of the MJO on the ocean and identify the nutrient source of Chl blooms in the MC. We found that the MJO could greatly enrich an oligotrophic land-enclosed sea in the MC because the phytoplankton biomass, estimated using Chl concentrations in the central Banda Sea, increased by 20% during the MJO active phases. The unique geographical features of land–sea contrast (e.g., narrow waters between islands elongated east to west) in the MC region are favorable for inducing strong ocean flow advection in the Karimata Strait, Java Sea, and Flores Sea and down to the Banda Sea. MJO wind–induced anomalous flow over the oceans changes the nutrient supply and, therefore, possibly also the biology of the nutrient-poor Banda Sea. As our results show, MJO-induced rainfall over land can be a strong factor indirectly affecting phytoplankton living a thousand kilometers away through oceanic advection transport.

Deforestation and landcover changes have altered the amount of carbon stored in the MC^35,36^ and caused more serious surface scouring when heavy rainfall occurs. Sediment loads in Indonesia are increasing at a higher rate than in other tropical regions because of large-scale deforestation^37^. Also, ocean temperatures rising in the region at a faster rate than the global average could strengthen the already stratified mixed layer and further inhibit entrainment of nutrient-rich deep waters to the sunlit layer^38^. Recent studies have indicated enhanced MJO and rainfall in a future warming scenario^39,40^. The compound effect of these anthropogenic phenomena may significantly change nutrient concentrations in MC waters. In addition to identifying a new physical process modulating nutrients, our study provides a crucial means of understanding future changes in the oceanic biogeochemical cycle in the MC.

## Materials and Methods

### Satellite ocean color data: Chlorophyll-a and TSM

The data used in this study on Chl designated for case 2 waters and TSM were obtained from the Medium Resolution Imaging Spectrometer (MERIS) sensor (Supplementary Table S1). Chl and TSM were estimated on the basis of different inherent optical properties: absorption for Chl, and scattering for TSM^41^. The TSM and Chl products derived using this method have been specifically developed for case 2 waters (i.e., waters where inorganic particles dominate over phytoplankton) and examined for coastal waters in Europe as well as coastal waters west of Borneo^42^. Since the area of study expands from shallow to deep waters, data of Chl for case 1 waters (i.e. waters where phytoplankton dominates over inorganic particles) were also examined for validation (figure not shown). The enhancement signal of the Chl in the central Banda Sea is prominent with both case 1 and 2 waters.

### MJO relevance satellite data

Daily precipitation data from the Tropical Rainfall Measuring Mission (TRMM) were employed for calculating MJO relevance (Supplementary Table S1). Subsurface observations in equatorial waters are almost completely lacking; therefore, for surface wind velocity and other oceanographic conditions, we converted 6-hourly products from the Climate Forecast System Reanalysis (CFSR)^43,44^ of the National Centers for Environmental Prediction (NCEP) (Supplementary Table S1) into a daily dataset. Daily CFSR subocean temperatures and salinities were compared with those of the Research Moored Array for African-Asian-Australian Monsoon Analysis and Prediction (RAMA) in the tropical Indian Ocean^45^ and the Tropical Atmosphere Ocean/Triangle Trans-Ocean Buoy Network (TAO/TRITON)^45^ in the Pacific Ocean at a 25-meter water level. A comparison of CFSR ocean temperatures showed a high correlation and low root mean square error with regard to the *in situ* observations, whereas salinity values matched the observations less well. In addition, CFSR currents were validated against Ocean Surface Current Analysis (OSCAR)^46^ satellite–derived sea surface currents on the MJO timescale (Supplementary Fig. S2 and Table 1). The variability of CFSR currents was well reproduced, but the OSCAR data had larger velocity amplitude.

### Wheeler and Hendon daily MJO index

The variables used in this study were those describing strong MJO conditions during northwest monsoons (December–February) from 2002 to 2010. MJO events in this analysis were defined using the Wheeler and Hendon^47^ daily MJO index, applying a 30–60 bandpass filter based on the first two principal components of the combined 850-hPa and 200-hPa zonal wind, and Outgoing Longwave Radiation (OLR) averaging 15°S–15°N. The daily OLR data were from the National Oceanic and Atmospheric Administration (NOAA), and the 200-hPa and 850-hPa zonal wind data from NCEP Reanalysis-2. Following the Wheeler and Hendon definition, an MJO life cycle was divided into eight phases, and all references to “phase” in this paper refer to diagnostics performed in the eight MJO phases (numbered I to VIII), composited using Wheeler and Hendon’s method. These phases represent the MJO’s propagation in both time and space. The interval between two phases was 5–7 days. This study only analyzed strong MJO events with an MJO index amplitude greater than two standard deviations and lasting longer than 30 days. The four years with strong events chosen for compositing were 2002, 2003, 2005, and 2007 (from December to the following February). MJO plots for each event are shown in Supplementary Figs S4–S9. To demonstrate the consistency among the cases, all composite analyses in this study were also performed on all MJO events from 2002–2010 (i.e., an MJO index amplitude greater than one standard deviation); the results are provided in Supplementary Figs S3, S8 and S9.

### MJO-scale anomaly retrieval

MJO anomalous values were obtained by applying a 30–60-weight Lanczos bandpass filter to the daily data after removing the climatological daily mean. Satellite data such as Chl and TSM, however, were not spatially interpolated or temporally continuous due to scanning swath gaps. We followed the procedure proposed by Jin *et al*.^19^. Instead of applying the bandpass filter directly, time-continuous MJO signals for Chl and TSM at each grid point were obtained by calculating first a 60-day and then a 30-day moving average of the data, then subtracting the 30-day moving average from the 60-day moving average. The daily MJO anomalies in each phase were then averaged to represent the fluctuation of atmospheric and oceanic conditions over the MJO life cycle^47^.

## Supplementary information

Supplementary Materials

## Data Availability

All data analyzed during this study are included in this published article and listed in Supplementary Table.
