# Supplementary material for: Madden–Julian Oscillation Enhances Phytoplankton Biomass in the Maritime Continent
Source: Sci Rep. 2019 Apr 1;9:5421. doi: 10.1038/s41598-019-41889-5 (PMC6443672; doi:10.1038/s41598-019-41889-5)
Supplement: Supplementary file 1 — Supplementary Materials [file 41598_2019_41889_MOESM1_ESM.pdf]

Supplementary Materials of

Madden–Julian Oscillation Enhances Phytoplankton Biomass in the Maritime Continent

Chiung-Wen June Chang, Huang-Hsiung Hsu*, Wee Cheah, Wan-Ling Tseng and Li-Chiang Jiang

Variable	Resolution	Data Source
Chlorophyll-a for type II water	0.25°×0.25°	European Space Agency (ESA) GlobColour http://www.globcolour.info
Total Suspended Matter (TSM)		
Photosynthetically Available Radiation(PAR)		
10m Surface Wind	0.3°×0.3°	Climate Forecast System Reanalysis (CFSR) http://cfs.ncep.noaa.gov/cfsr/
Ocean Current	0.5°×0.5°	
Outgoing Longwave Radiation (OLR)	2.5°×2.5°	National Oceanic and Atmospheric Administration (NOAA) http://www.esrl.noaa.gov/psd/
Surface Precipitation	0.25°×0.25°	Tropical Rainfall Measuring Mission (TRMM) http://trmm.gsfc.nasa.gov
15m Ocean Current	1/3°	Ocean Surface Current Analysis (OSCAR) https://podaac.jpl.nasa.gov/dataset/OSCAR_L4_OC_third-deg

Supplementary Table S1. Data sources used in this study.

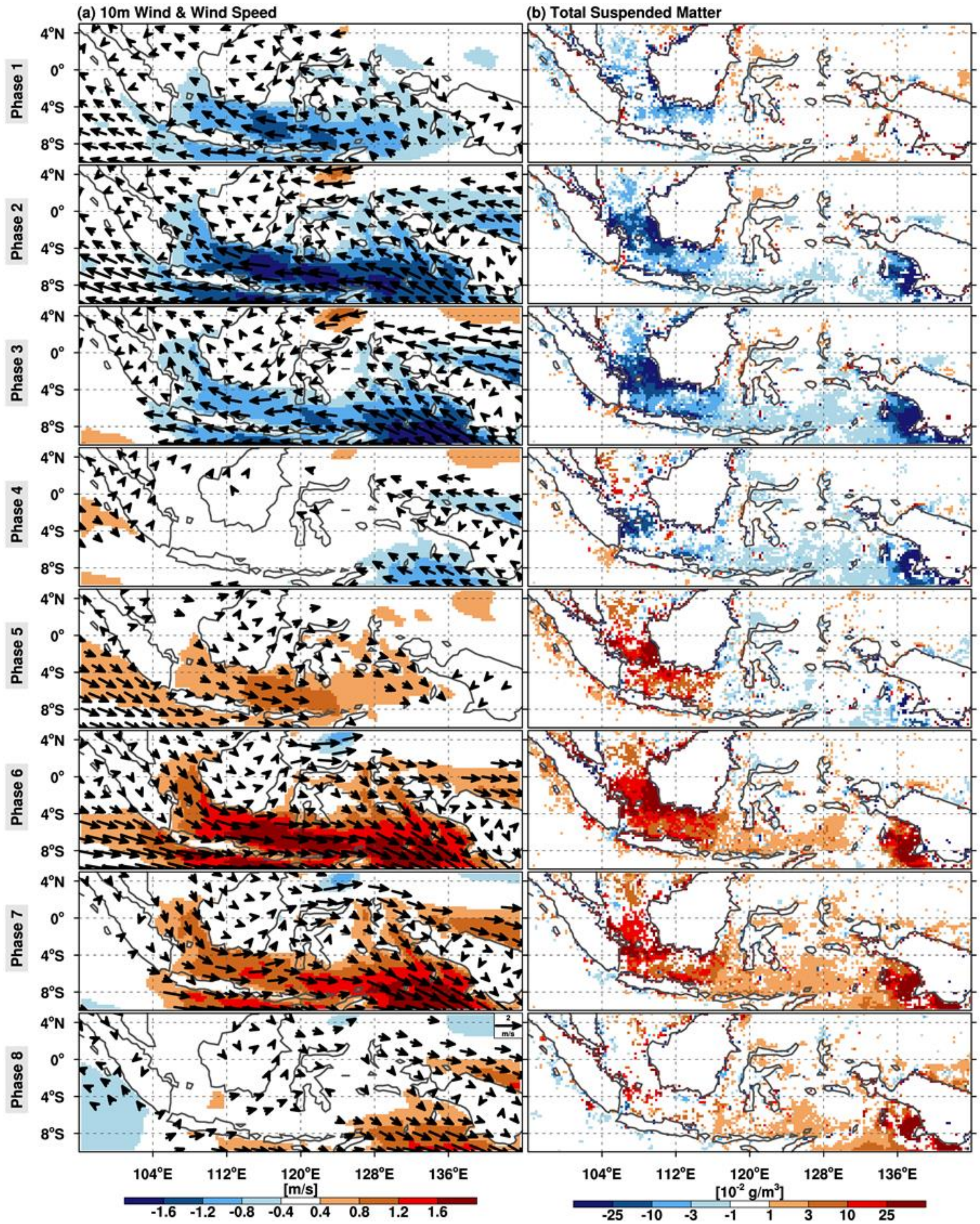

Supplementary Figure S1. Variable anomalies associated with the eight MJO phases: a) 10-m surface wind (shading) overlaid with wind direction (vectors); and b) TSM concentration.

Positive (negative) values indicate evidence of enhanced (suppressed) variables during the given phase. Only patterns significant at the 0.05 level are plotted.

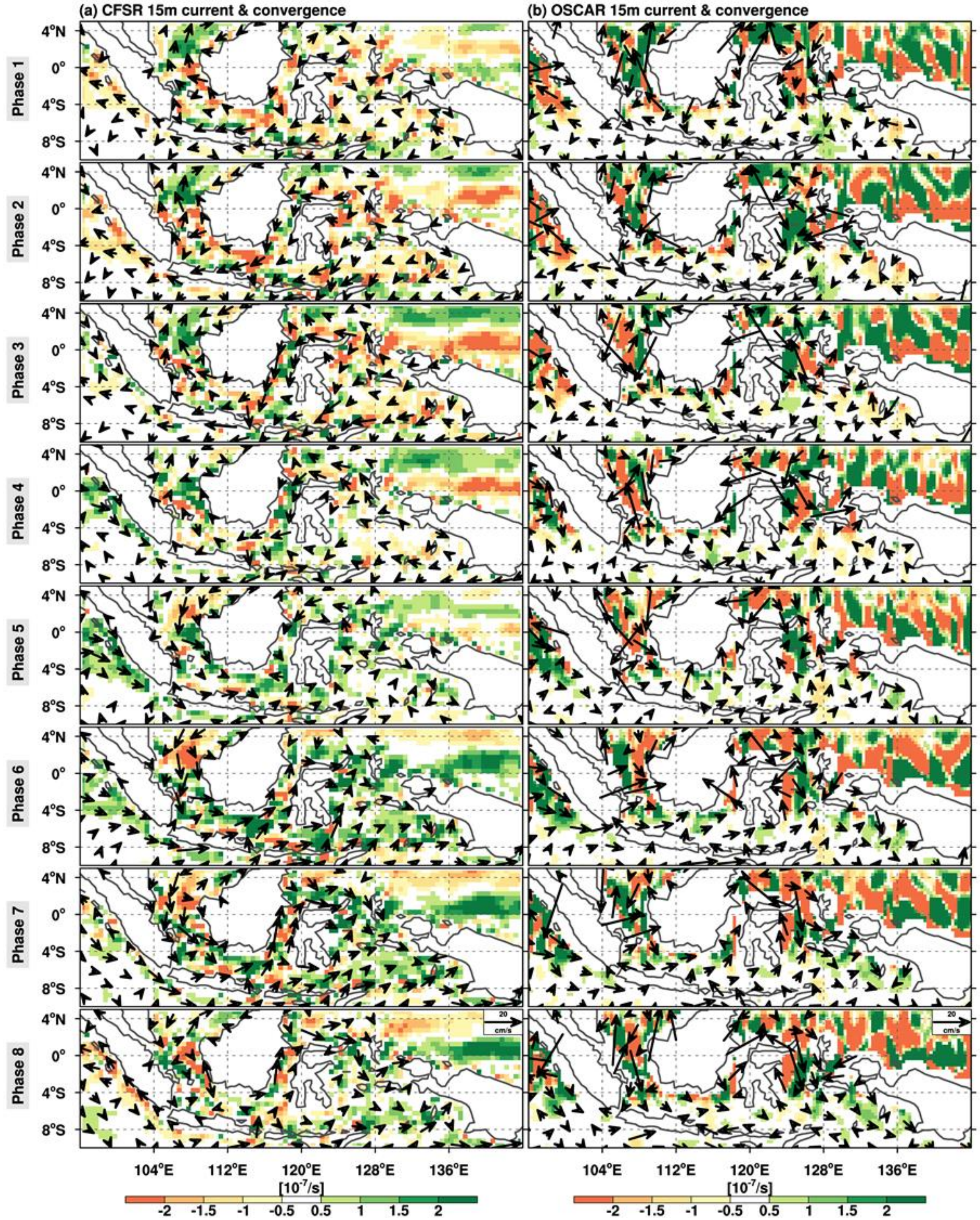

(a) CFSR 15m current & convergence
(b) OSCAR 15m current & convergence
Phase 1
Phase 2
Phase 3
Phase 4
Phase 5
Phase 6
Phase 7
Phase 8
4°N
0°
4°S
8°S
104°E
112°E
120°E
128°E
136°E
20 cm/s
[10⁻⁷/s]
-2 -1.5 -1 -0.5 0.5 1 1.5 2

Supplementary Figure S2. Four strong MJO events in composite maps of anomalies of 15-m ocean currents (vectors) and surface-water convergence (shading) from CFSR (left) and OSCAR (right). For OSCAR, only a current speed higher than 30 cm/s is displayed in the vectors.

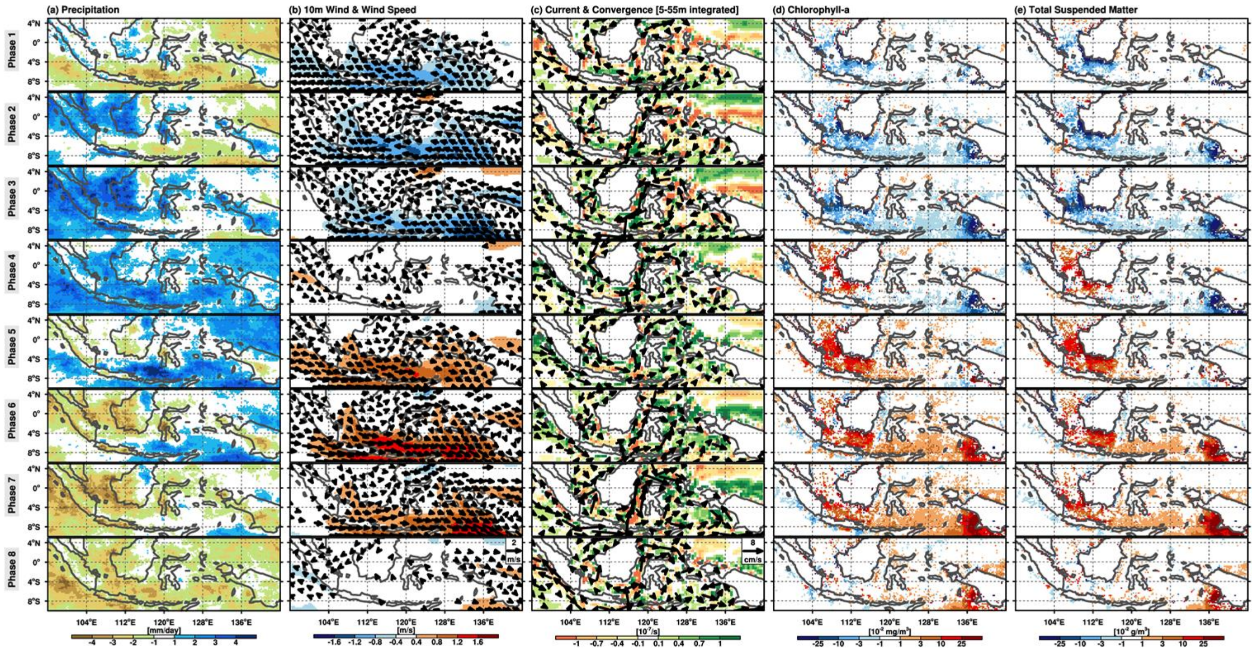

Supplementary Figure S3. Variable anomalies associated with the eight MJO phases for all MJO events in 2002–2010: a) precipitation; b) 10-m surface wind (shading) overlaid with wind direction (vectors); c) 5–55 m integrated ocean surface-water convergence (shading) overlaid with ocean flow (vectors); d) Chl; and e) TSM concentration. Positive (negative) values indicate evidence of enhanced (suppressed) variables during the given phase. Only patterns significant at the 0.05 level are plotted.

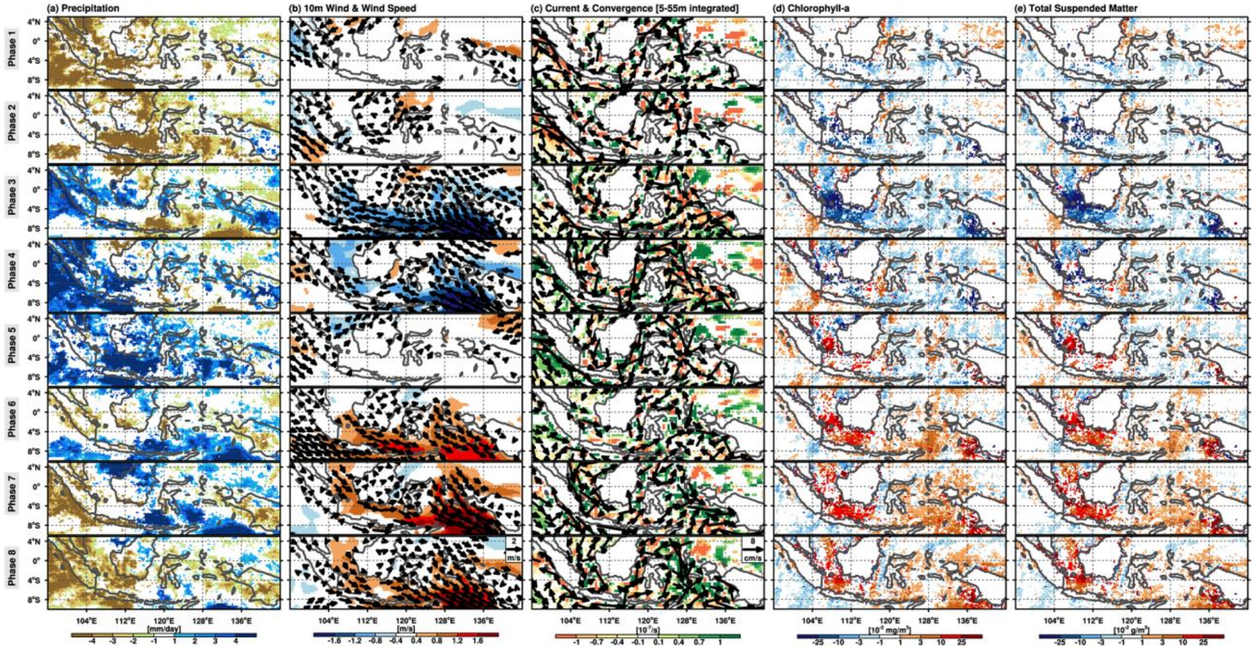

Supplementary Figure S4. Same as Supplementary Fig. S3, but only for the 2002 strong MJO event.

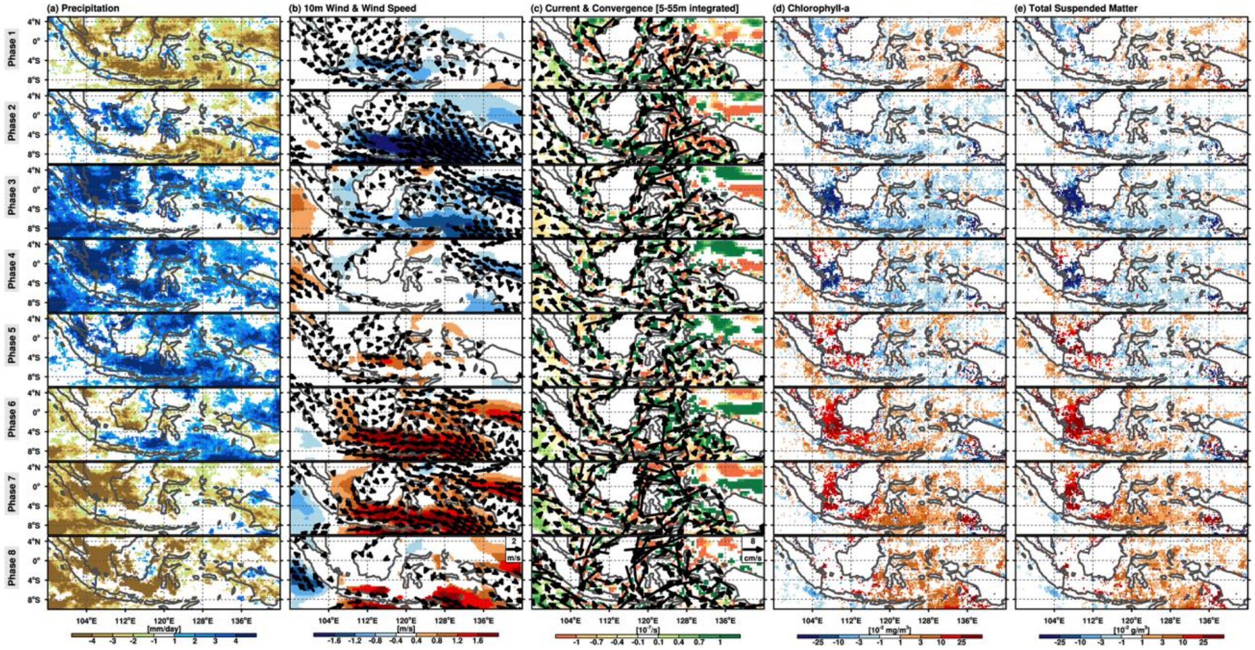

Supplementary Figure S5. Same as Supplementary Fig. S3, but only for the 2003 strong MJO event.

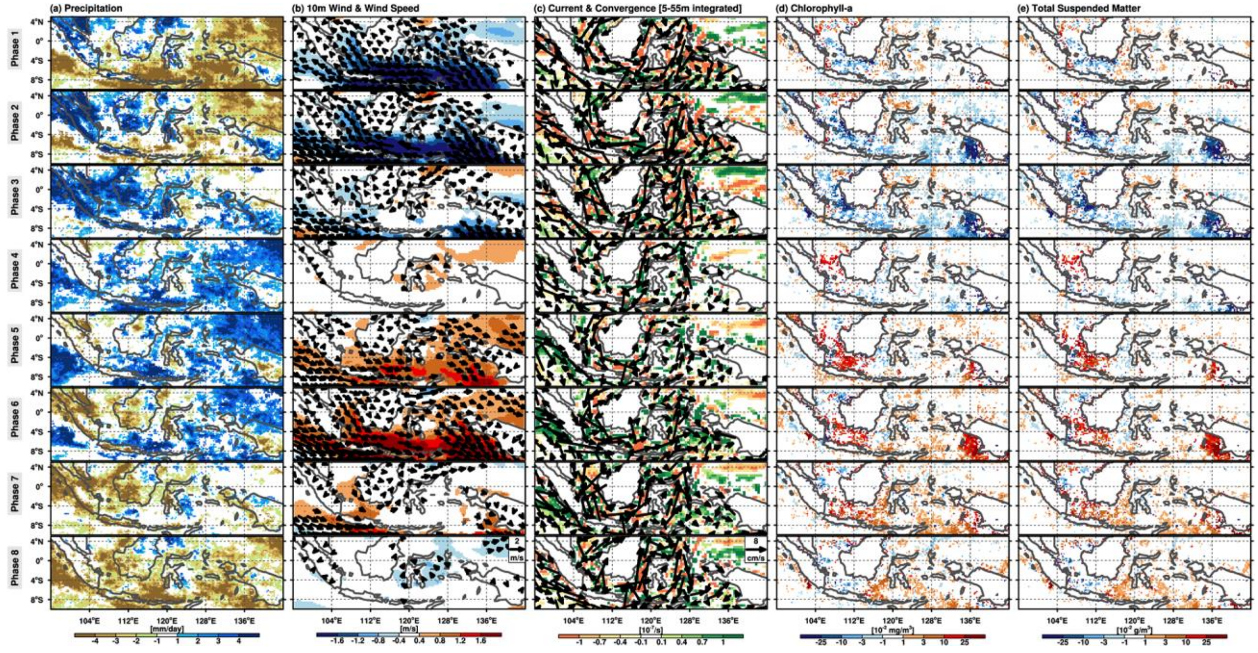

Supplementary Figure S6. Same as Supplementary Fig S3, but only for the 2005 strong MJO event.

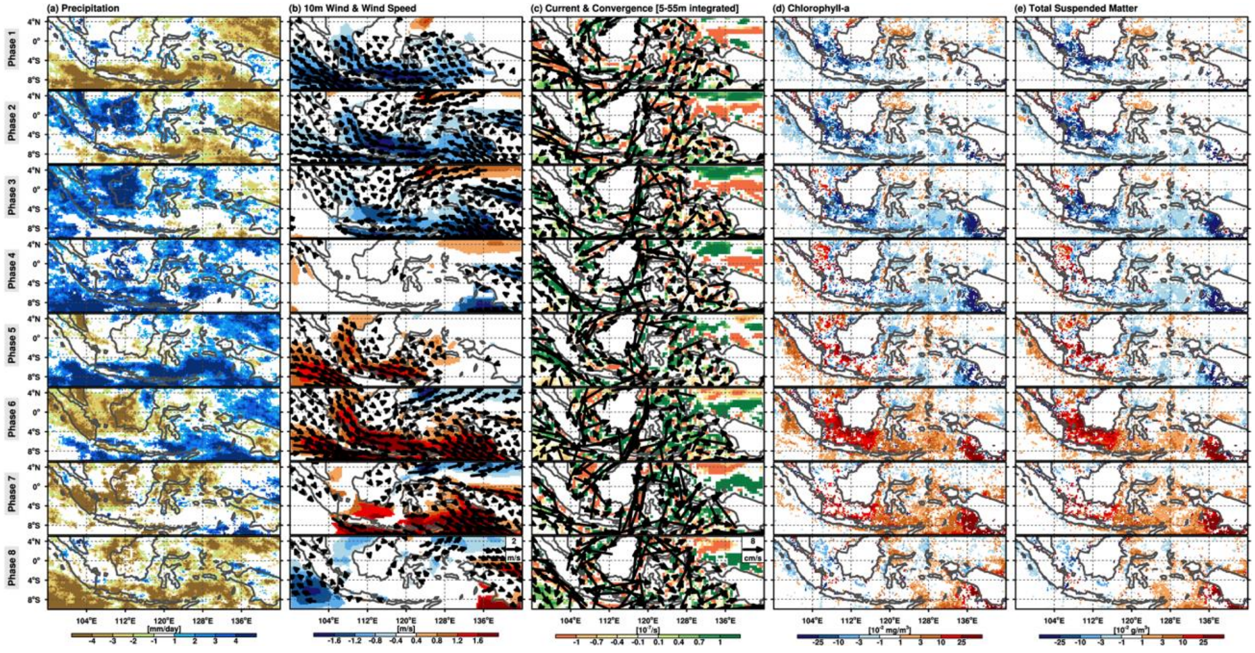

Supplementary Figure S7. Same as Supplementary Fig. S3, but only for the 2007 strong MJO event.

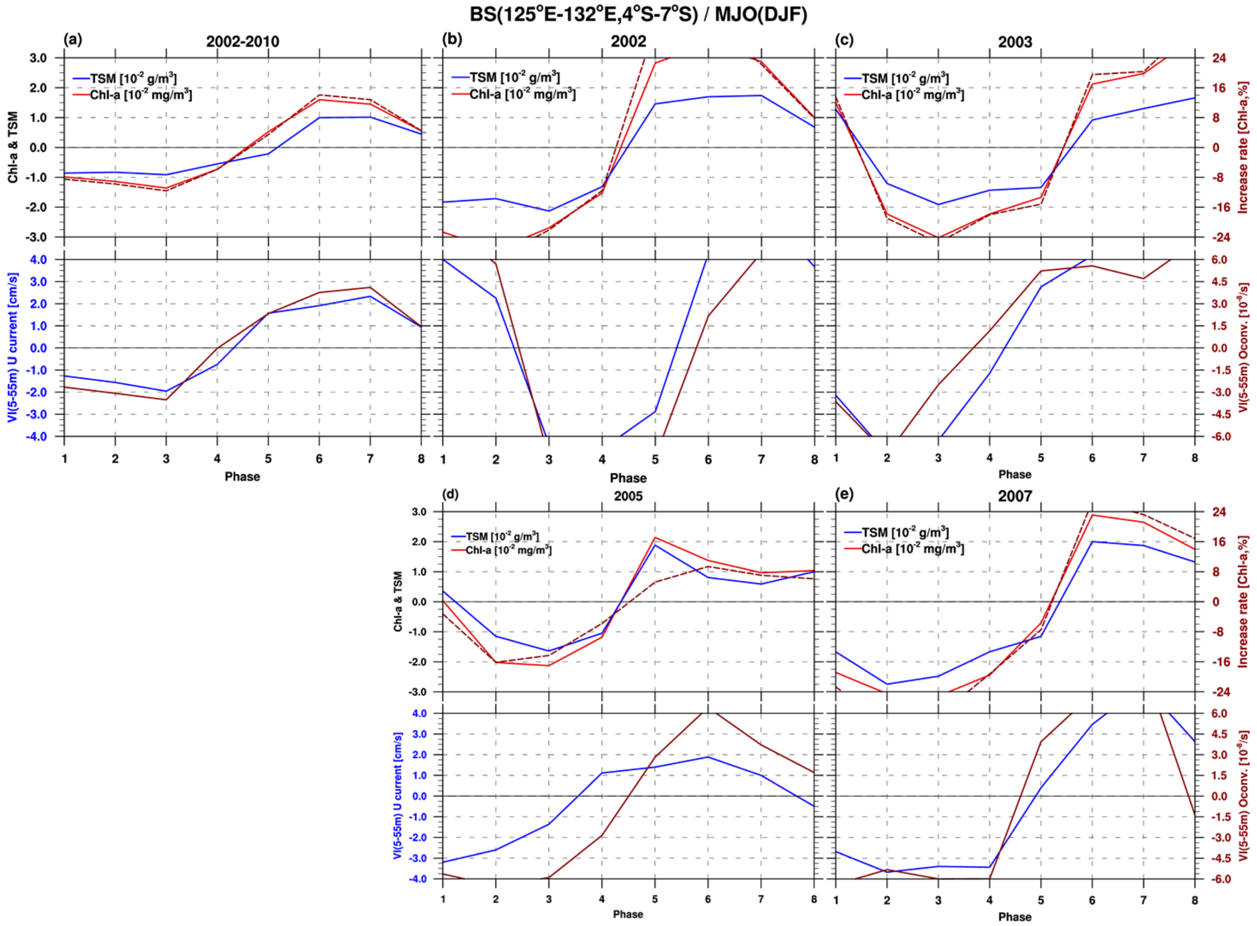

Supplementary Figure S8. Same as Fig. 4a, but for a) all MJO events during winter in 2002–2010, b) the 2002 strong MJO event, c) the 2003 strong MJO event, d) the 2005 strong MJO event, and e) the 2007 strong MJO event.

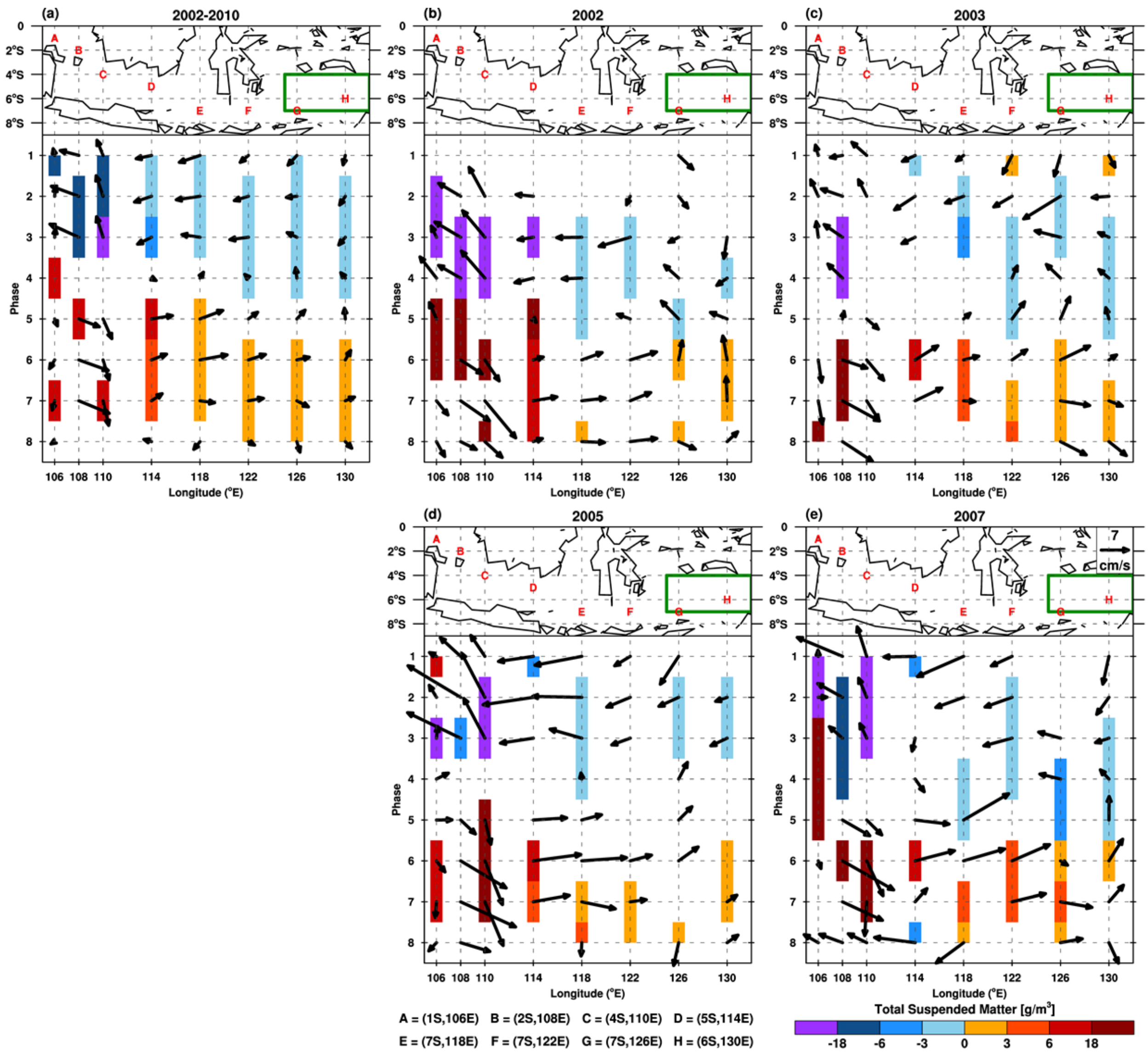

Supplementary Figure S9. Same as Fig. 5, but for a) all MJO events during winter in 2002–2010, b) the 2002 strong MJO event, c) the 2003 strong MJO event, d) the 2005 strong MJO event, and e) the 2007 strong MJO event. Here, the anomalous TSM and ocean flow vectors are plotted with every four longitude apart.
